# COGNITIVE FUNCTION AND ORAL HEALTH MEASURES AMONG OLDER ADULTS IN INDIA: EVIDENCE FROM THE LASI, 2017–2018

**DOI:** 10.1093/geroni/igae098.1631

**Published:** 2024-12-31

**Authors:** Uma Kelekar, Xi Pan, Muhammad T, Sidney Turner, Patricia Heyn, Bei Wu

**Affiliations:** Marymount University, Arlington, Virginia, United States; Texas State University, San Marcos, Texas, United States; The Pennsylvania State University, University Park, Pennsylvania, United States; Fors Marsh, Arlington, Virginia, United States; Marymount University, Fairfax Station, Virginia, United States; New York University, New York, New York, United States

## Abstract

Given the rising older adult population and related increase in the prevalence of cognitive impairment in India, the present study aimed to examine the association between cognitive function and oral health measures among the middle-aged and older adults in India. Data for the study were selected from the Longitudinal Aging Study in India (LASI, 2017-2018) and 56,723 respondents>=45 years were included. Oral health was measured by self-reported oral health problems including number of oral diseases, edentulism (i.e., losing some or all natural teeth), and dental care utilization (e.g., dental visit in the past 12 months). Our outcome measure was a composite score of cognitive function measured across five broad domains, specifically memory, orientation, arithmetic function, executive function, and object naming. Negative binomial regression models were used to analyze the relationship between the oral health and cognitive function. Our results showed that complete edentulism was associated with 3% lower cognitive scores [IRR=0.97, 95% CI 0.96, 0.99], while dental care utilization was positively associated with cognitive function. Those who had visited a dentist in the past 12 months reported 6% [IRR=1.06, 95% CI 1.03, 1.08] higher cognition scores than those who had not visited a dentist. The unadjusted univariate regressions demonstrated a negative association between the number of oral diseases and cognitive function. Our findings suggest an urgency for improving the existing oral health infrastructure, including expanding access to dental care and promoting oral health education in India, especially for adults at higher risk for cognitive impairment.

